# Accelerated tomotherapy delivery with TomoEdge technique

**DOI:** 10.1120/jacmp.v16i2.4964

**Published:** 2015-03-08

**Authors:** Sonja Katayama, Matthias F Haefner, Angela Mohr, Kai Schubert, Dieter Oetzel, Juergen Debus, Florian Sterzing

**Affiliations:** ^1^ Department of Radiation Oncology University Hospital Heidelberg Heidelberg; ^2^ Heidelberg Institute for Radiation Oncology (HIRO) Heidelberg; ^3^ Department of Radiation Oncology German Cancer Research Center Heidelberg Germany

**Keywords:** helical tomotherapy, TomoEdge, dynamic jaws, running‐start‐stop, beam‐on time

## Abstract

TomoEDGE is an advanced delivery form of tomotherapy which uses a dynamic secondary collimator. This plan comparison study describes the new features, their clinical applicability, and their effect on plan quality and treatment speed. For the first 45 patients worldwide that were scheduled for a treatment with TomoEdge, at least two plans were created: one with the previous “standard”mode with static jaws and 2.5 cm field width (Reg 2.5) and one with TomoEdge technique and 5 cm field width (Edge 5). If, after analysis in terms of beam on time, integral dose, dose conformity, and organ at risk sparing the treating physician decided that the Edge 5 plan was not suitable for clinical treatment, a plan with TomoEdge and 2.5 cm field width was created (Edge 2.5) and used for the treatment. Among the 45 cases, 30 were suitable for Edge 5 treatment, including treatments of the head and neck, rectal cancer, anal cancer, malignancies of the chest, breast cancer, and palliative treatments. In these cases, the use of a 5 cm field width reduced beam on time by more than 30% without compromising plan quality. The 5 cm beam could not be clinically applied to treatments of the pelvic lymph nodes for prostate cancer and to head and neck irradiations with extensive involvement of the skull, as dose to critical organs at risk such as bladder (average dose 28 Gy vs. 29 Gy, Reg 2.5 vs. Edge 5), small bowel (29% vs. 31%, Reg 2.5 vs. Edge 5) and brain (average dose partial brain 19 Gy vs. 21 Gy, Reg 2.5 vs. Edge 5) increased to a clinically relevant, yet not statistically significant, amount. TomoEdge is an advantageous extension of the tomotherapy technique that can speed up treatments and thus increase patient comfort and safety in the majority of clinical settings.

PACS numbers: 87.55.de, 87.55ne

## I. INTRODUCTION

**Figure 1:**
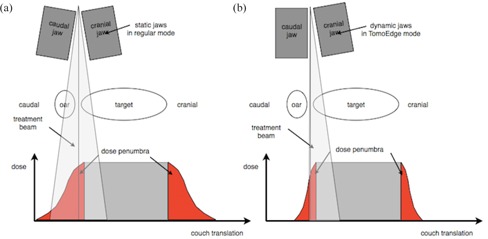
“Regular” tomotherapy vs. TomoEdge. Schematic illustration of “regular” tomotherapy delivery (a) and TomoEdge delivery (b) at the caudal end of a target. Note the reduced dose penumbra in TomoEdge mode. (Adapted from Sterzing et al.^(6)^).

As an advanced and highly versatile form of rotational intensity‐modulated radiotherapy (IMRT), tomotherapy has been in clinical use for almost a decade, enabling radiation oncologists to treat complex targets with excellent sparing of organs at risk such as, but not limited to, craniospinal irradiation,^(1)^ whole abdominal irradiation,^(2)^ and complex head and neck treatments.^(3)^ The technical details of tomotherapy have been described before.^(4)^ In brief, the tomotherapy treatment unit consists of a linear accelerator mounted on a ring gantry through which the treatment couch is moved during treatment. The treatment beam is modulated by a binary multileaf collimator, and the beam width is determined by the opening width of the secondary collimator, the so‐called “jaws”. Until the introduction of TomoEdge, which was called Running‐Start‐Stop in earlier publications^(5)^ and was already described in the initial publication on tomotherapy,^(4)^ the beam width remained fixed for the duration of a treatment (see Fig. 1(a)). Thus, a complete field width was irradiated at the cranial and caudal end of the target. The TomoEdge technology enables the superior and inferior jaw to open and close independently at the start and end of a target in order to reduce the longitudinal penumbra. This is done by the jaws waiting with an asymetric 1 cm slit at the caudal portion of the beam for the target to reach the beam. Moving along with the cranial border of the target, the superior jaw opens to the full treatment field. While the center of the target is irradiated with the full field width, the inferior jaw will close sequentially at the caudal border of the target until a 1 cm slit is left at the cranial portion of the beam. This 1 cm opening at the beginning and end of irradiation is necessary to achieve dosimetric accuracy. Figure 1(b) demonstrates TomoEdge irradiation at the caudal part of a target. Dosimetry and quality assurance procedures for the dynamic jaws are beyond the scope of this paper and will be described in a separate publication.

Before the clinical introduction of TomoEdge, planning studies have explored the dosimetric possibilities of Dynamic Jaw/Dynamic Couch (DJDC) mode, a research delivery mode that uses fully dynamic jaws and dynamic couch speed during the whole treatment delivery. Of note, the TomoEdge technique is part of the DJDC mode, but DJDC goes beyond the versatility of TomoEdge. For nasopharyngeal cancer, it could be demonstrated that DJDC with a 5 cm beam (DJDC 5) reduced the dose penumbra and consecutively the integral dose to normal tissue compared to regular delivery with a 2.5 cm and 5 cm field width (Reg 2.5 and Reg 5), respectively. Beam‐on time could be lowered by about 66%.^(6)^ Likewise, the treatment of very large target volumes, such as hemithoracic irradiation for mesothelioma, whole abdominal irradiation, and total marrow irradiation, with in DJDC 5 mode took less than half the time than the Reg 2.5 mode. With DJDC 5, integral dose was lower than with Reg 2.5 and Reg 5, reaching statistical significance in the case of whole abdominal irradiation.^(7)^

TomoEdge has been introduced in the University Hospital of Heidelberg, Germany in March 2013. This study explores the possibilities opened by this new delivery mode in clinical practice and highlights the shift from Reg 2.5 to TomoEdge with 5 cm field width (Edge 5) as the “standard” treatment mode.

## II. MATERIALS AND METHODS

### A. Patient population

The first 45 patients worldwide treated with TomoEdge were evaluated, thus the patient population contained a broad variety of cases. Nine patients received either definitive or postoperative irradiation for prostate cancer and four received adjuvant breast irradiation. Definitive radiochemotherapy for anal cancer was administered in three cases, while three patients received neoadjuvant radiochemotherapy for rectal cancer and one patient reirradiation for a presacral recurrence of rectal cancer. Malignancies of the head and neck region were treated in 16 cases: five patients underwent definitive radiochemotherapy, four received postoperative radiotherapy, and seven patients were treated with a combined modality irradiation with photon IMRT and a carbon ion boost. Radiotherapy of intrathoracic targets was performed in five patients: two were treated for lung cancer and three for esophageal cancer. The remaining five patients were irradiated at various locations, including partial brain irradiation and palliative treatment of bone metastases. For further detail, see Table 1.

**Table 1:** Localization and dose prescription for the 45 TomoEdge treatments

*Treatment*	*Dose to Target (Gy)*	*Dose to Boost (Gy)*	*Fractions*	*No. of Patients*	*Delivery Technique*
*prostate (9 patients)*					
prostate	76.5	–	34	2	Edge 5
pelvic lymph nodes+SIB to prostate	51	76.5	34	1	Edge 2.5
pelvic lymph nodes+SIB to prostate bed	45	54	18	4	Edge 2.5
pelvic lymph nodes+SIB to macroscopic lymph node metastases	46.8	57.2	26	2	Edge 2.5
*breast (4 patients)*					
breast+SIB to tumor bed	50.4	64.4	28	2	Edge 5
breast+ipsilateral supraclavicular nodes	50.4	–	28	1	Edge 5
breast+ipsilateral supraclavicular nodes+SIB to tumor bed	50.4	64.4	28	1	Edge 5
*gastrointestinal tract (6 patients)*					
pelvic and inguinal lymph nodes+SIB to anal canal	45	55	25	2	1 Edge 2.5 1 Edge 5
pelvic lymph nodes and rectum presacral reirradiation	50.4	–	28	3	Edge 5
	39.6	–	22	1	Edge 5
*head and neck (16 patients)*					
cervical lymph nodes+SIB to pharyngeal/laryngeal tumor	57.6	70.4	32	5	Edge 5
cervical lymph nodes+SIB to tumor bed in temporal area	54	60	30	2	1 Edge 2.5 1 Edge 5
cervical lymph nodes and base of skull (+sequential carbon ion boost)	50	–	25	2	1 Edge 2.5 1 Edge 5
cervical lymph nodes and floor of mouth or base of skull or paranasal sinuses (+sequential carbon ion boost)	54	–	27	3	2 Edge 2.5 1 Edge 5
cervical lymph nodes and base of skull or parotid bed (+sequential carbon ion boost) ipsilateral cervical lymph nodes+SIB to bed of lymph node metastasis	56	–	28	2	Edge 5
	57.6	68	32	1	Edge 5
cervical lymph nodes+SIB to hypopharyngeal tumor bed	54	66	30	1	Edge 5
*chest (5 patients)*					
right hilus	50	–	25	1	Edge 5
left lower lobe	40	–	10	1	Edge 5
mediastinal lymph nodes+SIB to esophageal cancer	50.4	58.8	28	1	Edge 5
mediastinal lymph nodes and esophageal cancer	45	–	25	2	Edge 5
other localizations (5)					
large intracranial meningioma	57.6	–	32	1	Edge 2.5
large astrocytoma+SIB	48	60	30	1	Edge 2.5
transverse processus of Th 5	40	–	20	1	Edge 5
ribs+right gluteal region	45	–	15	1	Edge 5
base of skull	39	–	13	1	Edge 2.5

SIB=simultaneous integrated boost.

### B. Delivery techniques

For each of the 45 patients, one plan with fixed jaws and a field width of 2.5 cm, representing the routine configuration used before TomoEdge, and one with Edge 5 was calculated. After plan analysis, the treating physician decided whether the Edge 5 plan was clinically acceptable. If the Edge 5 plan was refused, another plan with Edge 2.5 was created and used for treatment.

### C. Planning rules

The planning was performed by the same person to avoid bias caused by different planners. Dose coverage of target and boost volumes was kept as similar as possible for the different plans, and dose to organs at risk had to be below the tolerance dose and in accordance with the QUANTEC recommendations (for further detail, see Table 2).

**Table 2:** General planning rules and constraints (for conventional fractionation)

*Structure*	*Planning Rule*
PTV / boost volume	100% of the PTV receives at least 95% of the prescription dose
PTV / boost volume	D1 maximum 107% of the prescription dose
*prostate cases*	
rectum	$V_{40\;G}<40\%\text{maximum dose}\leq \text{prescription dose}$
bladder	$V_{65\;\text{Gy}}<50\%$
small bowel	maximum dose<53 Gy
*breast cases*	
ipsilateral lung	$V_{20\;\text{Gy}}<20\%$
contralateral lung	average dose<5 Gy
contralateral breast	average dose<5 Gy
*gastrointestinal tract cases*	
small bowel	As low as possible
bladder	As low as possible
*head and neck cases*	
parotid glands	one gland spared:average dose<20 Gy both glands spared:average dose<25 Gy
optic nerves, chiasm	maximum dose<54 Gy
*chest cases*	
contralateral lung	Mean lung dose<15 Gy
heart	As low as possible

$V_{20\;\text{Gy}‐65\;\text{Gy}}=\text{volume exposed to}\;20-65\;\text{Gy}$.

### D. Plan evaluation

Metrics to assess the plan quality with respect to PTV were as follows: dose received by 99% (D99) and 1% (D1) of the volume and the conformity index (CI), defined as the volume of the outer contour covered by the 95% isodose divided by the PTV volume covered by the 95% isodose.^(8)^

Integral dose as a general measure for organ‐at‐risk exposure was defined as the product of the volume of the outer contour and the average dose to that volume.^(9)^ In addition, the modulation factor used for each plan (defined as the ratio of maximum leaf open time to the mean leaf open time of all leaves that open in one projection), gantry rotation period, average and maximum dose, as well as organ volumes exposed to various dose levels ($V_{20\text{Gy}}$, $V_{40\text{Gy}}$, etc.), were assessed. Statistical analysis was performed with a two‐sided *t*‐test.

The patients gave written informed consent. Since this planning study was performed on anonymized data and patient's treatment was not affected by the study, approval of an ethics committee was not necessary.

## III. RESULTS

### A. Delivery parameters

**Figure 2:**
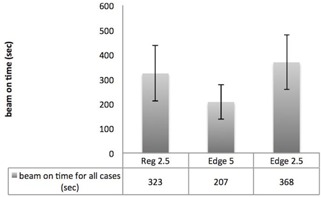
Beam‐on time. Average beam‐on time for Reg 2.5 and Edge 5 mode for all 45 cases. (Error bars represent±1 SD.)

As expected, the utilization of Edge 5 drastically reduced beam on time. Compared to Reg 2.5, average beam‐on time was 36% faster with Edge 5 for all plans (see Fig. 2). If only the cases are considered that actually received clinical treatment with Edge 5, beam‐on time was reduced by 35% (average beam‐on time 319 s vs. 209 s). The time reduction was statistically significant for prostate, breast, and head and neck cases, marginal for gastrointestinal cases (p=0.063), and not significant in the chest group and in the various locations group. As a consequence of the broader field width, the gantry rotation was slower, albeit not statistically significant, for the Edge 5 plans compared to Reg 2.5 plans (see Table 3). In contrast, the modulation factor used for the plans did not differ.

**Table 3:** Beam‐on time, average dose exposure of targets and healthy tissue, and plan characteristics for all cases and prostate, breast, and head and neck cases

	*Reg 2.5* ^a^	*Edge 5* ^a^	*p‐value*
*all cases*			
beam‐on time (s)	323±112	207±70	<0.001
D1 target (Gy)	58.52±9.95	58.85±9.90	0.878
D99 target (Gy)	45.92±7.98	45.54±7.60	0.817
standard deviation of average dose	±2.90	±2.97	–
Conformity Index	1.25±0.25	1.30±0.29	0.154
$V_{20\text{Gy}}$ external contour (%)	16.73±10.85	17.75±11.38	0.971
$V_{40\text{Gy}}$ external contour (%)	6.33±4.99	6.92±5.46	0.598
$V_{50\text{Gy}}$ external contour (%)	3.21±3.75	3.46±3.99	0.758
integral dose (Gy×1)	196±115	201±118	0.836
gantry period (s)	20.7±5.7	22.5±6.9	0.203
modulation factor	1.86±0.19	1.85±0.23	0.815
*prostate cancer*			
beam‐on time (s)	346±130	216±74	0.025
D1 target (Gy)	63.82±10.45	64.10±10.52	0.959
D99 target (Gy)	48.18±12.32	47.74±11.42	0.943
prostate only			
average dose rectum (Gy)	27.49	28.38	–
rectum $V_{40\text{Gy}}\;(\%)$	26.94	28.88	–
average dose bladder (Gy)	22.66	22.23	–
bladder $V_{40\;\text{Gy}}\;(\%)$	23.06	26.74	–
bladder $V_{50\text{Gy}}\;(\%)$	16.40	19.98	–
prostate cases with whole pelvis irradiation			
average dose rectum (Gy)	28.29±8.30	30.44±9.54	0.685
rectum $V_{40\text{Gy}}\;(\%)$	28.41±11.73	35.59±15.47	0.382
average dose bladder (Gy)	28.44±5.75	29.84±7.68	0.727
bladder $V_{40\;\text{Gy}}\;(\%)$	24.15±12.35	30.67±17.71 ^b^	0.474
bladder $V_{50\text{Gy}}\;(\%)$	11.24±6.23	12.61±9.13	0.789
small bowel $V_{20\text{Gy}}\;(\%)$	28.86±14.98	31.24±18.82 ^b^	0.812
small bowel $V_{40\text{Gy}}\;(\%)$	8.42±5.80	9.09±6.79	0.858
*breast cancer*			
beam‐on time (s)	362±41	222±11	0.001
D1 target (Gy)	62.40±5.30	62.43±4.83	0.994
D99 target (Gy)	43.86±0.66	43.55±0.99	0.665
average dose contralateral breast (Gy)	5.45±0.74	5.95±0.73	0.443
average dose ipsilateral lung (Gy)	13.71±0.82	13.72±1.45	0.990
$V_{20\;\text{Gy}}$ ipsilateral lung (%)	20.92±2.43	22.23±3.34	0.603
average dose contralateral lung (Gy)	5.28±0.81	5.46±0.85	0.800
*head and neck cancer*			
beam‐on time (s)	303±47	190±29	<0.001
D1 target (Gy)	63.10±7.27	63.29±7.52	0.944
D99 target (Gy)	49.59±3.96	49.34±3.74	0.859
average dose left parotid gland (Gy)	22.61±14.31	23.15±14.53	0.924
average dose right parotid gland (Gy)	18.51±17.64	19.20±17.19	0.921
maximum dose optic system (Gy)	16.92±18.11	16.41±18.72	0.942
average dose partial brain (only cases with skull involvement) (Gy)	18.98±5.44	21.22±5.83 ^b^	0.504

^a^ No statistically significant difference in dose exposure was detected between Reg 2.5 and Edge 5 plans.

^b^ Differences that were considered clinically relevant.

D1, D99=dose to 1% and 99% of the target volume; $V_{20‐50\text{Gy}}=\text{volume exposed to}\;20-50\;\text{Gy}$, respectively.

### B. Target coverage and exposure of organs at risk

Dose coverage of target and boost volumes could be maintained on a similar level in all delivery modalities; D1 and D99 for target volumes did not differ significantly between delivery modes. Dose conformity index and dose homogeneity, judged by average dose and its standard deviation, also were very similar (see Table 3).

Likewise, using a field width of 5 cm in Edge mode did not increase dose to healthy tissue as assessed by integral dose and exposure of the whole body ($V_{20\text{Gy}}$ to $V_{50\text{Gy}}$). For a detailed description, see Table 3. If only the cases for which the treating physician chose a 2.5 cm beam for clinical treatment were taken into account, the integral dose was higher in the Edge 5 plans compared to the plans with 2.5 cm field width. Yet, this difference did not reach statistical significance (Edge 5: 217 Gy× L, Reg 2.5: 199 Gy×L).

**Figure 3:**
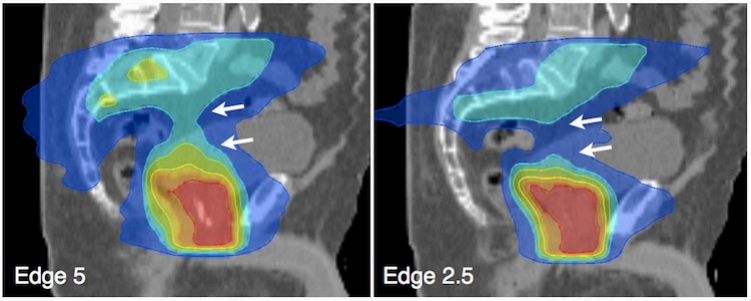
Pelvic irradiation. Example of treatment plans for prostate bed and pelvic lymph node areas, including the prescacral space S1‐3. The 5 cm beam resulted in a dose increase to healthy tissue (arrows) that was not deemed clinically acceptable.

Dose exposure of all examined organs at risk did not differ statistically significant between the different treatment modalities. Table 3 lists dose exposures of selected organs. However, some differences were considered clinically relevant. In prostate treatments which included the treatment of the pelvic lymph nodes, the 5 cm beam resulted in a higher exposure of the rectum and bladder on high dose levels ($V_{40\text{Gy}}$, $V_{50\text{Gy}}$), although this did not reach statistical significance (see Table 3). In plans with 2.5 cm field width, high‐dose areas could be shaped more precisely to the presacral lymphatic areas, which resulted in a better sparing of the bladder and the pelvic cavity (see Fig. 3).

**Figure 4:**
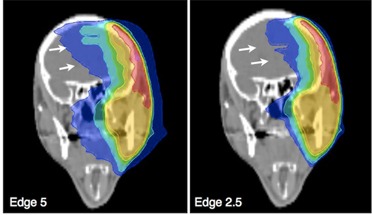
Head and neck irradiation. Example of head and neck treatment plans with skull involvement. The 5 cm beam resulted in a dose increase to the brain (arrows) that was not deemed clinically acceptable.

As shown in Fig. 4, in the case of head and neck targets that involved parts of the skull above the skull base, dose exposure of the brain was higher when Edge 5 was used (not significant). As consequence, Edge 2.5 was chosen as treatment modality in these cases.

### C. Consequences for clinical treatment

The modality chosen for treatment differed distinctly for the respective localizations: irradiation of the prostate alone could be performed with Edge 5 without hampering target coverage or organ at risk (OAR) sparing, while the use of a 5 cm beam substantially increased the dose to small bowel and bladder when the pelvic lymph nodes according to the RTOG consensus^(10)^ was included. Thus, Edge 2.5 was used. Pelvic treatment for rectal cancer was realized with Edge 5 in all cases, while the more complex target volume for anal cancer had to be treated with Edge 2.5 in one case. Since plan quality for breast cancer patients was equal for Reg 2.5 and Edge 5, the patients could be treated with the faster Edge 5 plan. Four of the 16 head and neck cancer patients received an Edge 2.5 plan because brain sparing was better than in the Edge 5 cm plan. The chest irradiations could all be performed with Edge 5 without compromises in OAR sparing. In total, in 30 of the 45 cases the Edge 5 mode could be applied.

## IV. DISCUSSION

In this first report on the clinical application of TomoEdge, it was demonstrated that this new tomotherapy delivery mode enables the planner to use a 5 cm field width in most cases. Among the first 45 cases treated with TomoEdge, two‐thirds were suitable for Edge mode with a 5 cm field width. The resulting reduction of beam‐on time may increase patient comfort, especially for treatments such as head and neck irradiations that require immobilization with thermoplastic masks. Additionally, shorter treatments potentially increase treatment accuracy and safety, as less intrafractional movement can occur. With the use of a 5 cm beam, a reduction of beam on time in the range of 50% would be expected.^(6)^ Yet, we could only reach a ~35% shorter beam on time as the gantry had to rotate slower to reach the same plan quality as with a smaller field width.

In our first clinical experience, we found two general exceptions to the applicability of Edge 5 mode: First, in irradiation plans of prostate and pelvic lymph nodes according to the RTOG consensus^(10)^ that included the presacral space while sparing rectum, small bowel, and bladder, the use of Edge 5 exposed larger parts of bladder and small bowel to high dose levels than a 2.5 cm field width (see Table 3). A similar problem occurred in cases of head and neck cancer with involvement of the skull that exceeded the base of skull: a 5 cm field width resulted in higher brain exposure than the 2.5 cm plan. In both pelvis and head and neck cases, the 2.5 cm plans were superior in reproducing the oblique shape of the target in longitudinal direction (e.g., along the sacral bone). The additional dose exposure was not statistically significant, but was deemed clinically relevant.

On the contrary, the 11 head and neck cases with targets just below the optic system could be treated very conveniently with Edge 5 without compromising the optic apparatus, as the maximum dose to the optic system was 7.07 Gy vs. 6.60 Gy (Reg 2.5 vs. Edge 5) for these cases.

The tomotherapy technique has always met with criticism concerning long beam‐on times.^11^, ^12^ However, with TomoEdge, treatment speed has reached the level of arc‐based treatment techniques, such as RapidArc (RA) and volumetric‐modulated arc therapy (VMAT) in many clinical situations.^(13)^ When comparing RA, VMAT, and tomotherapy plans for head and neck treatments, Van Gestel et al.^(14)^ reported beam‐on times of 3.05 min for RA, and 5.09 min for tomotherapy and VMAT. In our head and neck cases that were treated with Edge 5, we reached a very similar beam‐on time of 5.08 min for Reg 2.5 and a reduction to the level of RA (3.19 min) with Edge 5 mode. The same is true for the irradiation of localized prostate cancer — with an average beam‐on time of 2.46 min, Edge 5 treatments are as fast as single‐arc VMAT irradiations reported in literature.^(15)^

## V. CONCLUSIONS

The TomoEdge technique enables the user to apply a larger beam width in the majority of clinical cases and, as a consequence, to speed up treatments substantially. The percentage of clinical cases suitable for Edge 5 depends very much on the patient selection in the department. Even in the cases presented here, including head and neck cases with skull involvement and a high proportion of whole pelvis irradiations for prostate cancer, two‐thirds could be treated with Edge 5.

## ACKNOWLEDGMENTS

This work was supported by the Medical Faculty, University of Heidelberg, which provided a research fellowship for SK.
